# Ventilatory Support, Extubation, and Cerebral Perfusion Changes in Pre-Term Neonates: A Near Infrared Spectroscopy Study

**DOI:** 10.1089/neur.2023.0092

**Published:** 2024-04-11

**Authors:** Paolo Massirio, Valentina Cardiello, Chiara Andreato, Samuele Caruggi, Marcella Battaglini, Andrea Calandrino, Giulia Polleri, Federica Mongelli, Mariya Malova, Diego Minghetti, Alessandro Parodi, Maria Grazia Calevo, Domenico Tortora, Andrea Rossi, Luca Antonio Ramenghi

**Affiliations:** ^1^ Neonatal Intensive Care Unit, Maternal and Neonatal Department, IRCCS Istituto Giannina Gaslini, Genoa, Italy.; ^2^ Department of Neurosciences, Rehabilitation, Ophthalmology, Genetics, Maternal and Child Health (DINOGMI), and University of Genoa, Genoa, Italy.; ^3^ Epidemiology and Biostatistic Unit, Scientific Direction, and IRCCS Istituto Giannina Gaslini, Genoa, Italy.; ^4^ Neuroradiology Unit, IRCCS Istituto Giannina Gaslini, Genoa, Italy.; ^5^ Department of Health Sciences (DISSAL), University of Genoa, Genoa, Italy.

**Keywords:** cerebral perfusion, extubation, germinal matrix hemorrhage, intraventricular hemorrhage, NIRS, pre-term

## Abstract

Early extubation is considered to be beneficial for pre-term neonates. On the other hand, premature extubation can cause lung derecruitment, compromised gas exchange, and need for reintubation, which may be associated with severe brain injury caused by sudden cerebral blood flow changes. We used near infrared spectroscopy (NIRS) to investigate changes in cerebral oxygenation (rScO_2_) and fractional tissue oxygen extraction (+) after extubation in pre-term infants. This is a single-center retrospective study of NIRS data at extubation time of all consecutive pre-term neonates born at our institution over a 1-year period. Comparison between subgroups was performed. Nineteen patients were included; average gestational age (GA) was 29.4 weeks. No significant change was noted in rScO_2_ and cFTOE after extubation in the whole population. GA and germinal matrix hemorrhage (GMH)-intraventricular hemorrhage (IVH) showed a significant change in rScO_2_ and cFTOE after extubation. A significant increase in cFTOE was noted in patients with previous GMH-IVH (+0.040; *p* = 0.05). To conclude, extubation *per se* was not associated with significant change in cerebral oxygenation and perfusion. Patients with a diagnosed GMH-IVH showed an increase in cFTOE, suggesting perturbation in cerebral perfusion suggesting further understanding during this challenging phenomenon. Larger studies are required to corroborate our findings.

## Introduction

Extubating a pre-term infant is a challenging decision, especially during the first few days of life when there is a high risk of germinal matrix hemorrhage (GMH)-intraventricular hemorrhage (IVH). GMH-IVH is the most frequent lesion affecting infants born before the gestational age (GA) of 34 weeks, with incidence rates as high as 20–30%,^1^ and with variable outcomes. Most severe forms of GMH-IVH can affect neurological outcomes; also, a minor degree of hemorrhage may have a significant impact.^2–4^ Pathogenesis of IVH is multifactorial, and is closely linked to vascular immaturity within the germinal matrix and fluctuation of cerebral blood flow (CBF).^5,6^ Pre-term neonates with low GA typically exhibit impaired capacity for autoregulation of CBF.^7,8^ These neonates are particularly sensitive to CBF fluctuations, especially in cases of respiratory distress,^9^ pneumothorax,^10^ and mechanical ventilation, which is a known risk factor for GMH-IVH.^11^

Early extubation is considered to improve neurological outcomes of pre-term neonates.^12^ Prolonged mechanical ventilation exposes the pre-term infant to several adverse outcomes, including a higher risk of developing bronchopulmonary dysplasia^13^ and neurodevelopmental impairment.^14^ On the other hand, premature inappropriate extubation can cause lung derecruitment, compromise gas exchange, and increase respiratory fatigue, necessitating reintubation.^15^ Reintubation may potentially increase hemodynamic perturbations, especially those affecting CBF.^16^ This phenomenon may be aggravated by multiple attempts at reintubation, which is known to increase the risk of developing severe GMH-IVH.^17^

Near infrared spectroscopy (NIRS) is a potential tool to monitor the hemodynamic status in the neonatal brain. This technology can allow for continuous and non-invasive monitoring of cerebral tissue oxygenation (rScO_2_), even in most vulnerable infants.^18^ Starting from cerebral rSO_2_ and peripheral saturation (SpO_2_), it is possible to calculate the cerebral fractional tissue oxygen extraction (cFTOE), which is a better index of CBF than rScO_2_, especially in cases of low arterial oxygen levels (SpO_2_).^19^ The role of NIRS is still uncertain in pre-term neonatal intensive care, but is used as clinical practice in other contexts such as pediatric cardio-surgery monitoring; however, many studies have demonstrated that NIRS is useful for detecting different alterations that are potentially associated with brain injury, and it is widely used in many neonatal intensive care units (NICU).^20,21^ In our unit, NIRS has been part of standard monitoring in pre-term infants in the first hour of life since 2019. The levels of rScO_2_ and cFTOE depend on many variables such as blood pressure, patent ductus arteriosus (PDA), and anemia; in addition, these indices especially depend on respiratory factors such as SpO_2_, pCO_2_, and intrathoracic pressure,^22^ which are strictly linked to the ventilation mode.^23^

In this observational study, we evaluate any post-extubation changes in cerebral oxygenation and perfusion in a pre-term population.

## Methods

We retrospectively reviewed all consecutive patients who were monitored using NIRS at our institution between October 2021 and October 2022. The inclusion criteria were: GA <33 weeks, need for ventilatory support (either nasal continuous positive airway pressure [CPAP] or endotracheal tube [ETT]) at birth (within the first 5 h of life) and withdrawal of ventilatory support within the first 120 h of life (i.e., 5 days).

The decision to stop the ventilatory support (i.e., extubation) was in accordance with the institutional protocol based on ventilatory parameters; namely, peak inspiratory pressure (PIP) ≤21 cm H_2_O, positive end expiratory pressure (PEEP) ≤6 cm H_2_O, an FiO_2_ ≤ 25%, and good respiratory drive. In all cases, the extubation maneuver was performed by maintaining the NIRS sensor in place, and aspiration of secretions from the endotracheal tube was allowed at the discretion of the treating physician. Following extubation, CPAP or BiPAP was used as non-invasive ventilatory support based on the discretion of the treating physician.

**FIG. 1.**
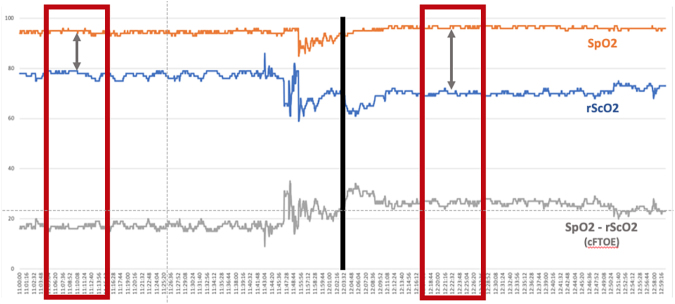
Squares show the time frame selected for the analysis. Vertical line represents the extubation time.

All patients underwent NIRS monitoring as per internal protocol using the MASIMO ROOT system (Masimo Corporation, Irvine, CA, USA); the MASIMO Radical 7 device was used to monitor peripheral blood oxygen saturation (SpO_2_), whereas the MASIMO O3 sensor was used to monitor cerebral regional oxygen saturation (rScO_2_). The MASIMO O3 sensor provides readings every 2 sec. It has been approved for use in pediatric patients and has a reported a bias, standard deviation (SD), standard error (SE), and root mean squared error (RMSE) for rSO_2_ of 2.6%, 4.5%, 0.3%, and 4.3%, respectively, and a trend accuracy with a relative mean error of -1.4%, SD of 4.3%, SE of 0.2%, and RMSE of 3.9%.^24^ In all patients, the adhesive sensor for cerebral rScO_2_ monitoring was attached to the left frontoparietal region, and monitoring was started within the first 5 h of life. Peripheral SpO_2_ monitoring was performed in the right upper limb. For the current study, rScO_2_ and SpO_2_ data within 1 h before and 1 h after the extubation were collected. To minimize errors caused by movement artifacts, rScO_2_ and SpO_2_ continuous readings were averaged over a 10-min time frame within 1 h before and after extubation. Such a brief time frame and a similar approach were already validated in other studies.^25^ The chosen time frame was based on the best stability of the rScO_2_ data (lower SD), greater availability of data, and best proximity to the extubation. Readings pertaining to the 15 min immediately before and after extubation were excluded from the current analysis (Figure 1). In addition to rScO_2_ and SpO_2_, post-extubation change in rScO_2_ (ΔrScO_2_) and SpO_2_ (ΔSpO_2_) were also calculated (i.e., rScO_2_ and SpO_2_ after extubation - rScO_2_ and SpO_2_ before extubation, respectively), and cFTOE was calculated using the formula: SpO_2_ - ScO_2_) / SpO_2_. The cFTOE is a useful index for assessment of cerebral regional O_2_ extraction and perfusion, which are independent of the variations in peripheral SpO_2_.

Clinical charts of all patients were reviewed, and general demographics along with GA, birth weight, 1-min and 5-min Apgar scores, presence (and treatment) of PDA at 48–72 h, need for inotropic therapy, ventilatory parameters before and after extubation (i.e., PIP, PEEP, and FiO_2_) were collected. All patients underwent brain ultrasound examination as per the institutional protocol at days 1, 2, and 3 after birth, and once a week thereafter until 36 weeks of age. Further, all patients underwent cerebral magnetic resonance imaging (MRI) at term age with a 3 Tesla MR system using an internal protocol that includes T1, T2, and susceptibility weighted imaging (SWI) sequence, which is highly sensitive to hemosiderin deposits.^26^ Presence of GMH-IVH was reported, and graded according to Volpe classification.^27^ Patients were divided into subroups according to clinical characteristics that could influence cerebral perfusion (GA < 28 weeks, birth weight <1000 g, significant PDA in treatment, presence of GMH-IVH, extubation timing <24h, and need for inotropic therapy).^20^

### Statistical analysis

Continuous variables were expressed as average ± SD (range) unless stated otherwise; categorical variables were expressed as frequency (percentage). Pre- and post-extubation NIRS parameters were compared using the Wilcoxon test. Mann–Whitney *U* test was used for comparison between subgroups. *P* levels ≤0.05 were considered indicative of statistical significance. Data were analyzed using the SPSS Statistics software, version 23 (SPSS, Chicago, IL, USA) and Microsoft Office Excel 2016 Professional (Microsoft, Redmond, WA, USA).

The studies involving human participants were reviewed and approved by Giannina Gaslini Hospital, Genoa, Italy. Parental informed consent was required and obtained before recruitment.

## Results

Out of 46 newborns who underwent NIRS monitoring at our institution during the study period, 27 patients were excluded because they were not extubated during the monitoring period and 6 patients had movement artifacts. A total of 19 patients (14 males) were included in the current study. Mean GA at birth was 29.4 ± 2.39 weeks (range 24.9–32.6) and average birth weight was 1353 ± 472 g (range 680–2170). On ultrasound screening, there were five cases of GMH-IVH (26.3%), two infants with stage I hemorrhage limited to the germinal matrix (IVH I, 10.5%), and three infants with stage II IVH (15.8%). Minor lesions in the periventricular white matter were confirmed in all three patients with stage II IVH on MRI performed at term-corrected age; SWI sequences (SWI + periventricular white matter lesions [PWML]) were positive for the presence of hemosiderin deposits in all three infants. All IVH cases were confirmed on ultrasound screening prior to extubation. A significant PDA requiring treatment was observed in six patients (31.6%). PDA was treated with paracetamol in three cases, ibuprofen in two cases, and both drugs in one case; none of the patients underwent surgical treatment. None of the patients were treated with inotropes (Table 1).

**Table 1.** Demographic and Baseline Characteristics of the Study Population

*n*	19
Sex	14 M, 5 F
Gestational age (weeks)	29.4 ± 2.4 (24.9–32.6)
Birth weight (g)	1352.9 ± 472.0 (680.0–2170.0)
Apgar score 1 min	5.8 ± 1.9 (1–9)
Apgar score 5 min	7.3 ± 1.1 (5–9)
IVH/GMH	5 (26.3%)
IVH > 1st degree	3 (15.8%)
GMH (%)	2 (10.5%)
No. of significant PDA on extubation (%)	5 (26.3%)
No. of treated PDA (%)	6 (31.6%)
Treatment with inotropes (%)	0 (0.0%)
ETT removal timing (h)	62.5 ± 37.4 (9.0–120.0)
Pre ETT removal	
PIP	16.6 ± 1.6 (14.0–21.0)
PEEP	4.8 ± 0.5 (4.0–5.6)
FiO_2_	0.22 ± 0.15 (0.21–0.25)
Post ETT removal	
PEEP	5.4 ± 0.6 (4.5–6.5)
FiO_2_	0.24 ± 0.05 (0.21–0.40)
Failed ETT removal	1 (5.3%)

Data are expressed as average ± standard deviation (range) unless stated otherwise.

IVH, intraventricular hemorrhage; GMH, germinal matrix hemorrhage; ETT, endotracheal tube; PIP, peak inspiratory pressure; PEEP, positive end-expiratory pressure; FiO2, fraction of inspired oxygen.

On average, extubation was performed at 62 h of life (range 9–120) according to a pre-defined set of parameters. Pre-extubation parameters were as follows: average PIP 16.6 cm H_2_O (range 14–21), average PEEP 4.7 cm H_2_O (range 4–5.6), and average FiO_2_ 22% (range 21–25). Following extubation, all patients received CPAP ventilation (in 4 cases with BiLevel option) with an average PEEP of 5.4 cm H_2_O (range 4–5.6) and an average FiO_2_ of 24% (range 21–40). There was only one case of failed extubation in our cohort (Table 1).

NIRS monitoring data are summarized in Table 2. Mean pre-extubation regional cerebral O_2_ saturation was 72.9% ± 5.4 (range 61.2–82.2) and did not change significantly after extubation (73.7% ± 7.6, range 56.5–85.3, *p* = 0.54). Likewise, there was no significant change in cerebral fractional tissue oxygen extraction index (cFTOE) after extubation (0.216 ± 0.053 vs. 0.219 ± 0.070; *p* = 0.82).

**Table 2.** NIRS Data for the Whole Study Population

	Before ETT removal	After ETT removal	Δ	*p* ^*^
rScO_2_ (%)	72.9 ± 5.4 (61.2–82.2)	73.7 ± 7.6 (56.5–85.3)	0.7 ± 5.2 (-7.8–11.9)	0.536
SpO_2_ (%)	93.6 ± 2.8 (88.3–97.7)	94.2 ± 3.1 (88.5–99.2)	0.6 ± 3.6 (-7.0–8.1)	0.507
cFTOE	0.216 ± 0.053 (0.140–0.358)	0.219 ± 0.070 (0.097–0.403)	0.003 ± 0.054 (-0.120–0.097)	0.816

Data are expressed as average ± standard deviation (range) unless stated otherwise.

^*^ *p* values from Wilcoxon test.

NIRS, near infrared spectroscopy; rScO_2_, regional cerebral oxygen saturation; SpO_2_, peripheral oxygen saturation; cFTOE, cerebral fractional tissue oxygen extraction; ETT, endotracheal tube; Δ, after ETT removal – before ETT removal.

The subgroup analysis showed significant data only in GA and GMH-IVH groups (Tables 3 and 4). Patients were divided into two groups according to their GA (≤ 28 weeks, group 1; > 28 weeks, group 2) and their GMH-IVH status (GMH-IVH, group 1; no GMH-IVH, group 2). A negative Δ rScO_2_ was observed in newborns with smaller GA compared with older infants (−4.6 ± 3.4 vs. 2.2 ± 4.7, *p* = 0.01), although no significant difference was noted in terms of Δ cFTOE between the two groups (*p* = 0.06). On the other hand, infants with IVH showed a larger increase in ΔcFTOE after extubation than infants without IVH (0.040 ± 0.035 vs. −0.010 ± 0.055, *p* = 0.05).

**Table 3.** Subgroup Analysis

	GA <28 weeks (*n* = 4)			GA ≥28 weeks (*n* = 15)			
	Pre	Post	Δ	Pre	Post	Δ	*p*
rScO_2_ (%)	72.4 ± 3.8	67.8 ± 2.5	-4.6 ± 3.4	73.0 ± 5.9	75.2 ± 7.8	2.2 ± 4.7	**0.01**
SpO_2_ (%)	93.1 ± 3.6	90.9 ± 1.4	-2.2 ± 4.8	93.8 ± 2.7	95.1 ± 2.8	1.3 ± 3.0	0.18
cFTOE	0.208 ± 0.048	0.254 ± 0.289	0.046 ± 0.037	0.218 ± 0.056	0.210 ± 0.075	-0.008 ± 0.053	0.06

	IVH / GMH (*n* = 5)			No IVH / GMH (*n* = 14)			
	Pre	Post	Δ	Pre	Post	Δ	*p*
rScO_2_ (%)	69.7 ± 6.1	66.7 ± 6.1	-3.0 ± 4.4	74.1 ± 4.8	76.2 ± 6.6	2.1 ± 5.0	0.06
SpO_2_ (%)	93.2 ± 3.2	94.4 ± 3.8	1.1 ± 5.4	93.8 ± 2.8	94.1 ± 2.9	0.4 ± 3.0	0.62
cFTOE	0.252 ± 0.065	0.292 ± 0.062	0.040 ± 0.035	0.203 ± 0.044	0.193 ± 0.053	-0.010 ± 0.055	**0.05**

Boldface represents *p* < 0.05.

Data are expressed as average ± standard deviation unless stated otherwise.

Pre, before endotracheal tube removal; Post, after endotracheal tube removal; rScO_2_, regional cerebral oxygen saturation; SpO_2_, peripheral oxygen saturation; cFTOE, cerebral fractional tissue oxygen extraction; GA, gestational age; IVH / GMH, intraventricular hemorrhage/ germinal matrix hemorrhage.

**Table 4.** Subgroup Analysis

	GA ≤28 weeks (*n* = 4)				GA >28 weeks (*n* = 15)			
	Pre	Post	Δ	*p*	Pre	Post	Δ	*p*
rScO_2_ (%)	72.4 ± 3.8	67.8 ± 2.5	-4.6 ± 3.4	0.14	73.1 ± 5.9	75.2 ± 7.9	2.2 ± 4.1	0.11
SpO_2_ (%)	93.1 ± 3.6	90.9 ± 1.4	-2.2 ± 4.8	0.27	93.8 ± 2.7	95.1 ± 2.8	1.3 ± 3.1	0.19
cFTOE	0.208 ± 0.048	0.254 ± 0.029	0.046 ± 0.037	0.07	0.218 ± 0.056	0.210 ± 0.075	-0.008 ± 0.053	0.57

	IVH / GMH (*n* = 5)				No IVH / GMH (*n* = 14)			
	Pre	Post	Δ	*p*	Pre	Post	Δ	*p*
rScO_2_ (%)	69.7 ± 6.1	66.7 ± 6.1	-3.0 ± 4.4	0.22	74.1 ± 4.8	76.2 ± 6.6	2.1 ± 5.0	0.16
SpO_2_ (%)	93.2 ± 3.2	94.4 ± 3.8	1.1 ± 5.4	0.50	93.8 ± 2.8	94.1 ± 2.9	0.4 ± 3.0	0.58
cFTOE	0.252 ± 0.065	0.292 ± 0.062	0.040 ± 0.035	**0.04**	0.203 ± 0.044	0.193 ± 0.053	-0.010 ± 0.055	0.78

Data are expressed as average ± standard deviation unless stated otherwise.

Pre, before endotracheal tube removal; Post, after endotracheal tube removal; rScO_2_, regional cerebral oxygen saturation; SpO_2_, peripheral oxygen saturation; cFTOE, cerebral fractional tissue oxygen extraction; GA, gestational age; IVH / GMH, intraventricular hemorrhage; germinal matrix hemorrhage.

## Discussion

Transition from invasive mechanical ventilation to non-invasive ventilatory support is a critical event in the life of pre-term infants. Early extubation or extubation failure exposes pre-term neonates to the risk of apnea, hypercapnia, and need for reintubation. These factors can lead to acute fluctuations in cerebral perfusion and increase the risk of IVH.^9,28^ On the other hand, prolonged or excessive mechanical ventilation can lead to hypocapnia and vasoconstriction leading to cerebral hypoperfusion and inflammation of white matter and increased risk of periventricular leukomalacia (PVL) and impaired brain development.^29,30^ Several guidelines and clinical criteria have been developed to help clinicians decide on the correct timing of extubation, which is typically exclusively based on the analysis of respiratory difficulties. Nevertheless, the decision making is often challenging, and it is a matter of intense debate among neonatologists.

In our study, no significant changes in rScO_2_ and cFTOE were found after extubation when looking at the whole population. This finding confirms what was previously described by Ericksen and coworkers in a smaller population of 12 pre-term patients.^25^ Peripheral SpO_2_ levels remained substantially stable (i.e., between 91% and 95%) throughout the study period, demonstrating that the extubation maneuver was well tolerated.^31^ There was no post-extubation brain bleeding event and we recorded only one case of failed extubation, which had no clinical consequences for the patient. No correlation was detected between extubation timing and ΔrScO_2_ or ΔcFTOE, even in the setting of early extubation. Nevertheless, in patients with GA <28 weeks, extubation was associated with a significant decrease in regional brain SaO_2_ compared with infants with GA ≥28 weeks (ΔrScO_2_ −4.6 ± 3.4 vs. 2.2 ± 4.7, *p* = 0.01). However, there was no significant difference between the two groups with respect to cFTOE index. Decrease in rScO_2_ in extremely pre-term infants can be explained by impaired cerebral autoregulation in these patients. Wong and coworkers showed how tissue oxygenation index and mean arterial blood pressure have high correlation (as a sign of lack autoregulation) in patients with lower GA.^8^ Further, Roche-Labarbe and coworkers also observed lower levels of cerebral oxygenation during the first 7 weeks of life of infants with GA <31 weeks using frequency domain NIRS monitoring, and the same authors also reported lower blood flow index in infants with GA between 24 and 27 weeks.^32^ It is unlikely that this may be the result of an increase in cerebral metabolism, as brain metabolism has been shown to increase with GA.^33^

On the other hand, newborns with GMH-IVH showed a significant change in the cFTOE index following extubation (ΔcFTOE 0.040 ± 0.035 vs. −0.010 ± 0.055, *p* = 0.05). Increase in the cFTOE index can be considered to be a sign of decreased brain perfusion.^19^ Verhagen and coworkers demonstrated that pre-term infants with GMH-IVH or periventricular hemorrhagic infarction (PVHI) had lower rScO_2_ and higher cFTOE during the first 2 weeks after birth than infants without GMH-IVH. Lower rScO_2_ and higher FTOE occurred irrespective of the grade of GMH-IVH.^34^ This was confirmed by Lin and coworkers, who focused on low-grade GMH-IVH, and by Vesoulis and coworkers more recently.^35,36^ Because FTOE reflects the balance between cerebral oxygen supply and cerebral oxygen consumption, increased FTOE can be explained either by reduced oxygen supply or increased oxygen consumption. A lower oxygen supply may result from lower CBF.^32^ Our study highlighted worsening of the cFTOE index following extubation, which is a potential sign of further impaired CBF autoregulation in these patients.^7,33,37^ NIRS evaluates the oxygenation of most superficial cerebral tissue (∼ 2 cm of depth from the skin).^38,39^ GMH-IVH bleeding is located in the deep regions of the brain; therefore, this correlation suggests an influence on oxygenation and perfusion of more superficial areas of the brain, as previously demonstrated with MRI techniques by our group.^38^ Cerebral cortex and subcortical areas (e.g., subplate neurons) are very important for cerebral connectivity and neuroplasticity.^40–42^ This further effect on the frontal areas following extubation in infants already having GMH-IVH may have greater adverse effect on the development of local neural circuitry. In addition, if a similar effect is likely to happen also in the posterior part of the brain, where brain maturation is known to be more advanced than in the frontal part,^43,44^ impaired development of the visual cortex may also be postulated, although NIRS is unlikely to allow assessment of the posterior part of the brain for technical reasons. Vasospasm secondary to the increase in locally produced pro-inflammatory cytokines has been cited as the underlying mechanism for cerebral cortex hypoperfusion associated with GMH-IVH.^45^ This could be part of a complex pathophysiological pattern that includes other pathogenic noxae such as increase in pro-inflammatory factors and reduced cell proliferation of the germinative matrix.^46,47^ This may also help explain why even low-grade bleeding is associated with impaired neurological outcomes^3,4^ and impaired periventricular white matter maturation in pre-term infants.^2,48,49^ Further, the positioning of the NIRS sensor in our study was over the front-parietal portions of the brain that are known to exhibit delayed maturation compared with posterior portions.^50^ Reduction in CBF in these more immature areas could have a greater impact on brain maturation. At least, in our population, three out of the five patients with GMH-IVH also had PWML with a hemorragic appearance (positive on SWI at MRI), which are minor lesions of white matter.^51^ These lesions have been described to be more represented in cases of GMH-IVH,^51^ and may be correlated with altered venous blood flow and perfusion in these patients.

Some limitations of our study should be considered. The relatively small sample size may affect the generalizability of our results, in particular for subgroup analysis, where only a limited number of patients had GMH-IVH and low GA. Although rScO_2_ data are known to be affected by blood pressure variations, it is worth noting that no inotropes were used in our patients and that blood pressure values were stable throughout the study period. Finally, although we detected significant changes in the cFTOE index after extubation, our monitoring data were limited to few minutes after extubation.

## Conclusion

Our study shows that NIRS monitoring can help detect subclinical cerebral hypoperfusion events in pre-term infants. Our cohort confirms that extubation was not related to significant CBF fluctuation, even in the setting of early extubation. A significantly increased cerebral tissue extraction fraction of oxygen caused by reduction of brain perfusion was observed following extubation only in those patients with GMH-IVH. This is an important finding, as extubation can aggravate brain hypoperfusion described to follow GMH-IVH. Further, without NIRS monitoring, these hypoperfusion events would have been completely undetected because of the stability of the other parameters. These findings suggest the need for a more cautious approach when mechanically ventilated newborns with GMH-IVH are considered for extubation because of changes in ventilatory strategies or improvements in respiratory conditions. Larger studies are required to confirm these findings.

## Data Availability

All study data and materials will be made available upon written request to the corresponding author.

## Abbreviations Used

CBF: cerebral blood flow
cFTOE: fractional tissue oxygen extraction
CPAP: continuous positive airway pressure
ETT: endotracheal tube
GA: gestational age
GMH-IVH: germinal matrix hemorrhage-intraventricular hemorrhage
MRI: magnetic resonance imaging
NICU: neonatal intensive care unit
NIRS: near infrared spectroscopy
PDA: patent ductus arteriosus
PEEP: positive end expiratory pressure
PIP: peak inspiratory pressure
PWML: periventricular white matter lesions
RMSE: root mean squared error
SD: standard deviation
SE: standard error
SWI: susceptibility weighted imaging
